# Multiple statistical tests: Lessons from a d20

**DOI:** 10.12688/f1000research.8834.2

**Published:** 2016-09-07

**Authors:** Christopher R. Madan

**Affiliations:** 1 Department of Psychology, Boston College, Chestnut Hill, MA, 02467, USA

**Keywords:** statistical analysis, error, probability, statistical test

## Abstract

Statistical analyses are often conducted with α= .05. When multiple statistical tests are conducted, this procedure needs to be adjusted to compensate for the otherwise inflated Type I error. In some instances in tabletop gaming, sometimes it is desired to roll a 20-sided die (or 'd20') twice and take the greater outcome. Here I draw from probability theory and the case of a d20, where the probability of obtaining any specific outcome is ^1^/ _20_, to determine the probability of obtaining a specific outcome (Type-I error) at least once across repeated, independent statistical tests.

## Introduction

In scientific research, it is important to consider the issue of conducting multiple statistical tests and the likelihood of spuriously obtaining a ‘significant’ effect. Within a null-hypothesis significance testing (NHST) framework, statistical tests are usually conducted with *α* = .05, i.e., the likelihood of falsely rejecting the null hypothesis as .05. Interestingly, this value coincides with the probability of obtaining a specific outcome on a 20-sided dice (or ‘d20’), as $\frac{1}{20}$ = .05. In the current (fifth) edition of Dungeons & Dragons, a tabletop game, many in-game events are determined based on the outcome of a d20. However, to make some events more likely, there are times when players roll a d20 ‘with advantage’, meaning that they roll the d20 twice and take the greater value ^1^. (There are also instances where a d20 is rolled ‘with disadvantage’, where the lesser value is taken, but here I will only focus on the former case.) This parallels the use of NHST without any correction for multiple comparisons, as it is more likely to get a significant effect due to chance (i.e., Type-I error) if many tests are conducted without a correction for multiple comparisons.

Here I wondered how much the probability of obtaining a 20, on a d20, would increase due to multiple tests–i.e., obtaining at least one 20 across *n* die. This approach assumes that each statistical test is wholly independent from each other, and thus is likely to over-estimate the effect related to conducting multiple statistical tests using variations in how the measures are calculated or the use of different, but correlated, measures. Nonetheless, this exploration is based in probability theory and mathematical derivations, rather than computational simulations, and can serve as an comprehensible primer in understanding the relationship between repeated statistical tests and probability distributions.

Developing an intuition of statistics and probability distributions is of particular importance as most people, both laymen ^2^ and scientists ^3, 4^, have misconceptions about NHST. This is further compounded by critics of NHST, which often over-emphasize the limitations of the approach, e.g., see 4– 6. By providing a comprehensible example of how repeated statistical tests can inflate chance likelihoods, I hope that these demonstrations can improve researchers’ intuitions regarding NHST. This approach is not contrary to those suggested by the use of confidence intervals and Bayesian statistics—which have become increasingly adopted across the life sciences, from medicine to psychology ^7, 8^—but rather to improve comprehension of the characteristics of NHST.

## Mathematical derivations

The probability that of a specific outcome occurring on each on *n* die, each with *d* sides is:

$$P\left(d,n\right)=1–{\left(\frac{d–1}{d}\right)}^{n}$$

The probability of obtaining a specific outcome across *n* rolls of a *d*-sided die are listed in Table 1.

**Table 1.** Probability of obtaining a specific outcome at least once, using a *d*-sided die rolled *n* times.

*n*	*d* = 2	*d* = 6	*d* = 20	*d* = 100	*d* = 1000
1	.5000	.1667	.0500	.0100	.0010
2	.7500	.3056	.0975	.0199	.0020
3	.8750	.4213	.1426	.0297	.0030
4	.9375	.5177	.1855	.0394	.0040
5	.9688	.5981	.2262	.0490	.0050
6	.9844	.6651	.2649	.0585	.0060
7	.9922	.7209	.3017	.0679	.0070
8	.9961	.7674	.3366	.0773	.0080
9	.9980	.8062	.3698	.0865	.0090
10	.9990	.8385	.4013	.0956	.0100
20	1.0000	.9739	.6415	.1821	.0198
50	1.0000	.9999	.9231	.3950	.0488
100	1.0000	1.0000	.9941	.6340	.0952
500	1.0000	1.0000	1.0000	.9934	.3936
1,000	1.0000	1.0000	1.0000	1.0000	.6323
10,000	1.0000	1.0000	1.0000	1.0000	1.0000

To develop some intuition of the effect of multiple die rolls, several simple cases can be considered.

For *d* = 2, i.e., a coin, the probability of obtaining at a heads when flipping one coin ( *n* = 1) is $\frac{1}{2}$. The probability of obtaining a heads twice (with two coins, *n* = 2) is ${\left(\frac{1}{2}\right)}^{2}$ or $\frac{1}{4}$. In contrast, the probability of obtaining at least one heads when flipping two coins is $\frac{3}{4}$, as there are four possible outcomes ({HH, HT, TH, TT}) and three of them satisfy the criteria of ‘at least one heads’ ({HH, HT, TH}) and only one outcome does not ({TT}). This can more clearly be considered as the complementary event, where the probability is 1 − $\frac{1}{4}$, which resolves to $\frac{3}{4}$.

For *d* = 6, i.e., a ‘conventional’ six-sided die, the probability of obtaining any specific outcome is $\frac{1}{6}$. When considering multiple dice, it is again important to differentiate the probability of obtaining ‘obtaining the same specific outcome multiple times’, e.g., the probability of obtaining two sixes with two dice is ${\left(\frac{1}{6}\right)}^{2}$ = $\frac{1}{36}$, from the case of ‘obtaining at least one specific outcome across multiple dice’. To determine the probability of obtaining a specific out-come on *any* of multiple dice, the complementary event should again be considered, i.e., the probability of not obtaining that outcome on any of the die. For *n* = 1, the probability of *not* obtaining a specific outcome is $\frac{5}{6}$. Following from this, the probability of obtaining that specific outcome is 1 − $\frac{5}{6}$ or $\frac{1}{6}$. When *n* = 2, the probability of not obtaining a six on either of the dice is ${\left(\frac{5}{6}\right)}^{2}$, which resolves to $\frac{25}{36}$. The complementary event of obtaining ‘at least one six’ is 1 − $\frac{25}{36}$ or $\frac{11}{36}$. Here we can see that with two dice, the probability of obtaining at least one six (or any other specific outcome) is nearly doubled, from $\frac{6}{36}$ (i.e., $\frac{1}{6}$ with a single die).

For *d* = 20, i.e., a 20-sided die, the probability of obtaining any specific outcome is $\frac{1}{20}$ or .05. If *n* = 2 dice are rolled, the probability of obtaining at least one 20 is $\frac{39}{400}$ or .0975. If *n* = 10 dice are rolled, the probability of obtaining at least one 20 is ≈ .4013. With *n* = 20 dice, this increases further to ≈ .6415.

We can also consider a more general problem, the probability of obtaining an outcome of *o* or greater, on at least one of *n d*-sided die:

$$P\left(d,n,o\right)=1–{\left(\frac{d–\left(d–o+1\right)}{d}\right)}^{n}$$

**Figure 1.**
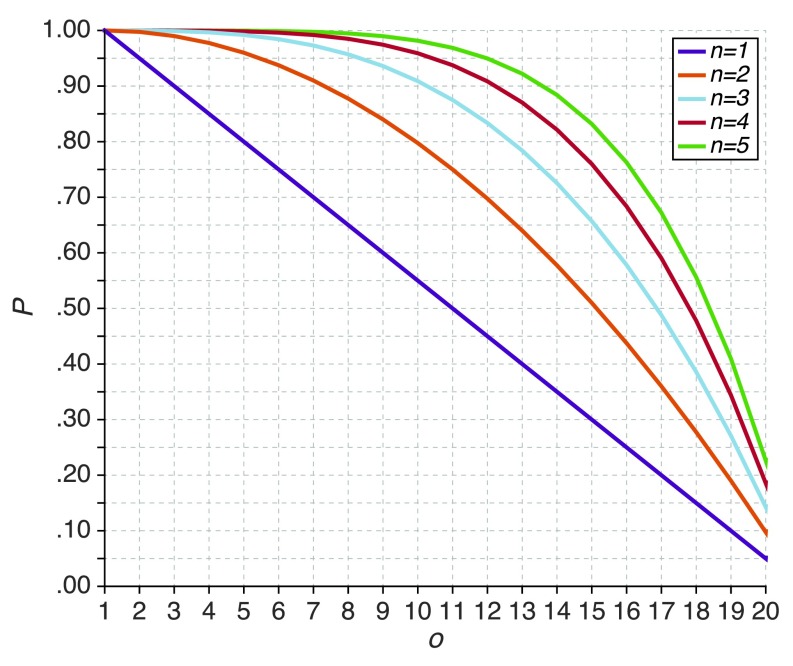
Probability ( *P*) of obtaining at least an outcome *o* once, across *n* d20 die.

For instance, when rolling a six-sided die, the probability of obtaining a five or higher is $\frac{2}{6}$ (equivalent to $\frac{12}{36}$). Following from the same approach of calculating the complementary event, the probability of obtaining not obtaining any two specific outcomes across multiple dice is 1 − ${\left(\frac{4}{6}\right)}^{2}$, which resolves to $\frac{20}{36}$. Figure 1 and Table 2 show the probability of obtaining at least *o* on a *d* = 20 die, across *n* dice.

**Table 2.** Probability of obtaining at least an outcome *o* once, across *n* d20 die.

*o*	*n* = 1	*n* = 2	*n* = 3	*n* = 4	*n* = 5
1	1.0000	1.0000	1.0000	1.0000	1.0000
2	.9500	.9975	.9999	1.0000	1.0000
3	.9000	.9900	.9990	.9999	1.0000
4	.8500	.9775	.9966	.9995	.9999
5	.8000	.9600	.9920	.9984	.9997
6	.7500	.9375	.9844	.9961	.9990
7	.7000	.9100	.9730	.9919	.9976
8	.6500	.8775	.9571	.9850	.9947
9	.6000	.8400	.9360	.9744	.9898
10	.5500	.7975	.9089	.9590	.9815
11	.5000	.7500	.8750	.9375	.9688
12	.4500	.6975	.8336	.9085	.9497
13	.4000	.6400	.7840	.8704	.9222
14	.3500	.5775	.7254	.8215	.8840
15	.3000	.5100	.6570	.7599	.8319
16	.2500	.4375	.5781	.6836	.7627
17	.2000	.3600	.4880	.5904	.6723
18	.1500	.2775	.3859	.4780	.5563
19	.1000	.1900	.2710	.3439	.4095
20	.0500	.0975	.1426	.1855	.2262

## Discussion

While it is widely understood that multiple comparisons need to be corrected for, many would underestimate the degree of inflation in Type-I error associated with additional, uncorrected statistical tests. Critically, statistical procedures have been developed to correct for multiple comparisons (e.g., Bonferroni, Tukey’s HSD), see 9 for a detailed review. Nonetheless, the mathematical derivations presented here clearly illustrate the influence of multiple statistical tests on the likelihood of obtaining a specific outcome due to chance alone. These derivations and examples should be useful in providing a concrete example of the problem associated with uncorrected multiple comparisons and may prove useful as a pedagogical tool.
