# Supplementary material for: Multiple statistical tests: Lessons from a d20
Source: F1000Res. 2016 Sep 7;5:1129. Originally published 2016 Jun 2. [Version 2] doi: 10.12688/f1000research.8834.2 (PMC4902094; doi:10.12688/f1000research.8834.2)
Supplement: Peer review history

# Peer review: Referee response for version 2

**DOI:** 10.5256/f1000research.10322.r16168

**Published:** 2016-09-07

**Authors:** Jens Foell

**Affiliations:** 1 Department of Psychology, Florida State University, Tallahassee, FL, USA

I have read this submission. I believe that I have an appropriate level of expertise to confirm that it is of an acceptable scientific standard.

# Peer review: Referee response for version 1

**DOI:** 10.5256/f1000research.9509.r14147

**Published:** 2016-07-05

**Authors:** Steven R Shaw

**Affiliations:** 1 Department of Educational and Counselling Psychology (ECP), McGill University, Montreal, QC, Canada

The author provides an excellent foundation for developing an intuitive understanding of null hypothesis significant testing. The concept of using a 20 sided die to assist graduate students and new researchers to better understand what exactly is meant by .05 and how multiple comparisons have a dramatic influence on interpretation is an interesting one — and I believe novel. This intuitive approach can also be used to improve understanding for the general public and overall science communication. Although this report includes a mathematical derivation, which may be too advanced for new scholars or science communication, it provides an excellent rationale for the use of 20 sided die.

 When can also easily imagine that this rationale can be used better understand robustness of the outcomes of studies that may be influenced by experiment-wise error rates, such as in the cases of multiple attempts at replication or multiple trials of a specific experiment. The 20 sided die provides a concrete and real world method of communicating the complexities of multiple comparisons that is far more user-friendly than random number generators and variations of Monte Carlo studies.

 I am curious as to whether the exactitude in engineering and manufacturing a 20 sided die will result in exactly equal probability of each number appearing. A six sided die is created with right angles and is relatively easy to create an equal probability of landing on each side. Obviously, this makes no difference or changes the point of the paper; yet, it may add error should anyone actually attempt to roll the 20 sided dice multiple times. Just a thought as I am unsure on this issue. I suppose should a 20 sided die found to contain significant error or even bias, then there would be scandal in the Dungeons & Dragons world.

 The author also deserves credit for establishing high levels of nerd credibility. When Dungeons & Dragons, mathematical derivations, and useful statistical communication methods are combined into a single published scientific paper; the trifecta of nerd credibility has been achieved.

 Overall, this is a well-written report and the mathematics is correct. I am hopeful that the author continues to elaborate on this concept and develops other uses of a 20 sided die for communication and lesson plans in courses on research design and basic applied statistics. This will also be helpful in explaining experiment wise error rate/multiple comparisons in the cases of science communication as well.

I have read this submission. I believe that I have an appropriate level of expertise to confirm that it is of an acceptable scientific standard.

# Peer review: Referee response for version 1

**DOI:** 10.5256/f1000research.9509.r14150

**Published:** 2016-06-08

**Authors:** Matthew Wall

**Affiliations:** 1 Division of Brain Sciences, Imperial College London, London, UK

This short report provides a simple and concise illustration of some of the issues surrounding multiple comparisons in statistical testing. I see nothing wrong with the logic or the mathematics, and can see that this would make a valuable contribution as an assigned text on an introductory statistics course.

 The only suggestion I have for material that could be added would be to include a citation to additional material on the topic of multiple-comparisons correction for the interested reader. This would make the piece more valuable as a teaching aid. I'd suggest adding a couple of sentences to the discussion along those lines and citing a review paper on the topic, such as Curran-Everett, 2000 ^1^

 Couple of minor points:

1. The paragraph after table 1 starts 'For intuition,'. This seems an odd phrase to me. Maybe replace with 'Intuitively' or 'For the purposes of familiarity...' or something like that.
2. Just before the general equation the phrase 'across n die' appears on its own, as a separate paragraph. An error? Or is this supposed to be a subtitle?

I have read this submission. I believe that I have an appropriate level of expertise to confirm that it is of an acceptable scientific standard.

## References

[1] Curran-EverettD: Multiple comparisons: philosophies and illustrations. *Am J Physiol Regul Integr Comp Physiol*.2000;279(1) :R1-8 1089685710.1152/ajpregu.2000.279.1.R1

# Peer review: Referee response for version 1

**DOI:** 10.5256/f1000research.9509.r14149

**Published:** 2016-06-03

**Authors:** Jens Foell

**Affiliations:** 1 Department of Psychology, Florida State University, Tallahassee, FL, USA

The author provides a novel demonstration of the increasing probability of spurious research results when performing multiple tests: noticing the equivalence of a p = .05 statistical threshold and a d20 die (as used in popular games), the author goes on to describe changes in probabilities when using multiple dice, or when allowing multiple sides of the die to count as a correct result. The aim of this article, as I understand it, is to demonstrate that these changes have a surprisingly large influence on statistical hypothesis testing, and at the same time to provide a hands-on example that many readers might be able to relate to (in the form of the d20 die).

 The article is well-written and in my opinion fulfills both of these goals. Its rationale and the mathematical derivations seem to be sound and correct. I can easily see this article being used by educators to teach the topic of spurious statistical results and to make the topic more accessible.

 I have some minor edits/recommendations to increase the overall clarity and readability of the article:

- When the game name "Dungeons & Dragons" is first mentioned in the introduction, it should be followed by a citation of the game's publisher, year of publication, and, if necessary/appropriate, copyright information.
- A d6 die is described as "regular" in the text. This term seems ambiguous to me and I recommend replacing it with a term such as "cube-shaped," "classical," or "conventional."
- The current version of the manuscript appears to contain a printing error: before the last paragraph of the "mathematical derivations" section, the sentence fragment "across n die." is printed without context.

I recommend the indexing of this article after these minor issues have been addressed, and I hope the author will continue to produce research notes that highlight statistical issues in an approachable manner.

I have read this submission. I believe that I have an appropriate level of expertise to confirm that it is of an acceptable scientific standard.
